# Torsades de Pointes and Prolonged Self-Terminating Ventricular Fibrillation Induced by Amiodarone

**DOI:** 10.7759/cureus.11693

**Published:** 2020-11-25

**Authors:** Georgios D Tziatzios, Matthaios Didagelos, Ioannis Tziatzios, Stavros Hadjimiltiades, Theodoros Karamitsos

**Affiliations:** 1 Cardiology, AHEPA Hospital, Aristotle University of Thessaloniki, Thessaloniki, GRC

**Keywords:** torsades de pointes, ventricular fibrillation, arrhythmia, amiodarone, prolonged qt

## Abstract

A 71-year-old man with a recent diagnosis of pneumonia developed paroxysmal atrial fibrillation and was admitted to the cardiology service. Amiodarone was administered intravenously to restore sinus rhythm. Significant prolongation of the QT interval (QTc = 640ms) was noted and an exceedingly prolonged (over 3 minutes), self-terminating, episode of ventricular flutter/fibrillation occurred during bedside monitoring. The event was terminated without first converting to a more organized ventricular rhythm and without any adverse neurological sequelae. Apart from the long duration of ventricular fibrillation and its spontaneous termination, our case highlights the importance of the continuous heart rhythm monitoring in patients with extreme QT interval prolongation.

## Introduction

Acquired QT interval prolongation may be induced by both cardiovascular and several non-cardiovascular drugs and can be exaggerated by multiple risk factors. Its incidence is difficult to be estimated and there is an inherent risk to predict the absolute risk for a given individual. A QTc (corrected QT) interval >440 ms is considered prolonged although arrhythmias are most often associated with values of ≥500 ms. Its clinical importance lies to the fact that it can be fatal, leading to Torsades de Pointes (TdP) and ventricular fibrillation [1].

## Case presentation

**Figure 1:**
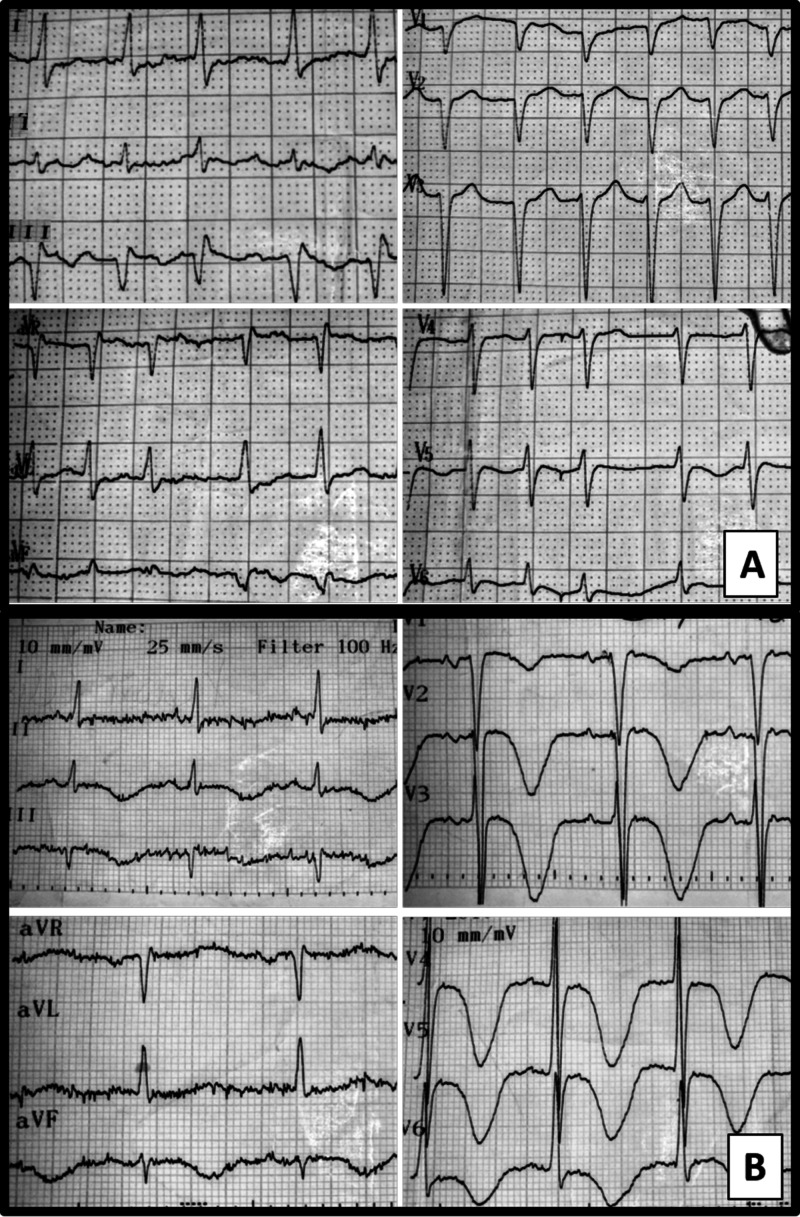
(A) Electrocardiogram (ECG) during atrial fibrillation; (B) ECG after conversion to sinus rhythm.

**Figure 2:**
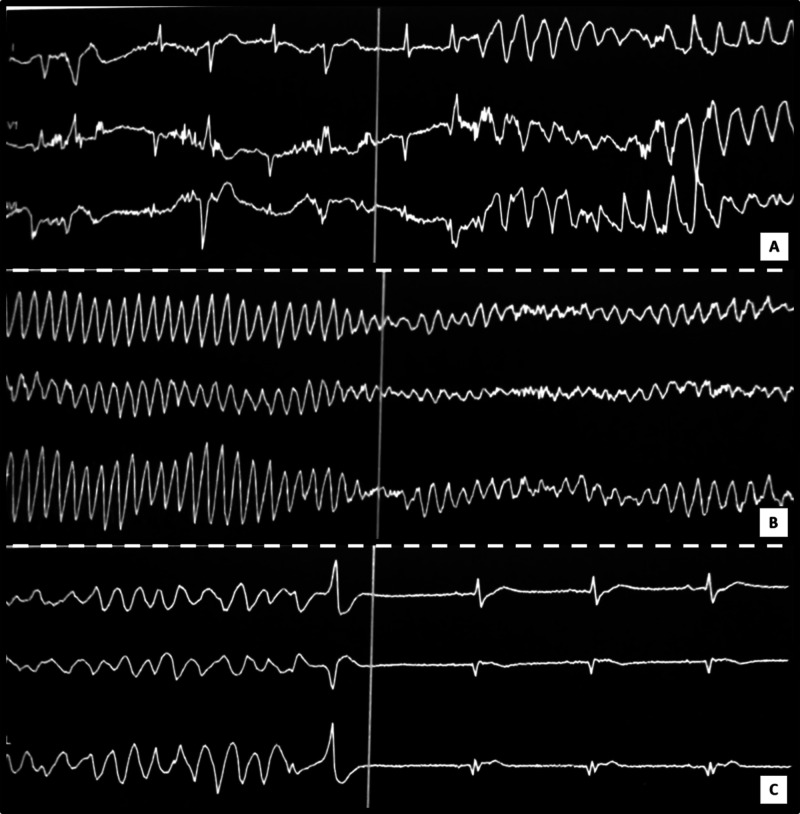
(A) Monitor recording with an “R on T beat” leading to Torsades de Pointes; (B) Ventricular flutter and ventricular fibrillation; (C) Spontaneous restoration to sinus rhythm.

A 71-year-old man with previous history of coronary artery disease, ischemic stroke and peripheral arterial disease, was readmitted 10 days post initial discharge, because of recurrent pneumonia, despite having been under antibiotic treatment (moxifloxacin per os). This second event was treated as a healthcare-associated pneumonia with a combination of antibiotics (intravenous tazobactam and clarithromycin). Forty-eight hours later, paroxysmal atrial fibrillation occurred (Figure 1A) and intravenous amiodarone (1800 mg total dose) was administered for 36 hours, until sinus rhythm was restored. After restoration of sinus rhythm, his electrocardiogram (ECG) showed extreme QT prolongation (QTc = 640ms) with deep T wave inversion in inferior and all precordial leads (Figure 1B), although no electrolytic abnormalities were noted. Continuous heart rhythm monitoring was initiated and all QT prolongating drugs (clarithromycin, amiodarone) were discontinued. A few hours later, a syncopal episode occurred. The rhythm on the monitor was ventricular fibrillation. While the defibrillator was being attached, the arrhythmia was self-terminated with no apparent neurological sequelae. Post hoc analysis of the monitor recording revealed an “R on T beat”, leading to TdP (Figure 2A) and soon degenerating to prolonged ventricular flutter and ventricular fibrillation (VF) (Figure 2B), which was restored spontaneously to sinus rhythm (Figure 2C). The entire episode lasted 3 minutes and 16 seconds (Video 1).

**Video 1:** Heart rhythm monitor recording of the whole event in the coronary care unit. Note the duration of the event.

Following this arrhythmic event, a 2g bolus of magnesium sulfate was administered intravenously and slow infusion of potassium was initiated in order to maintain serum potassium levels at the high-normal range [1]. Short-lived and self-terminating episodes of TdP were noticed, despite the aforementioned treatment. Given that the patient’s heart rate was relatively slow (55-60 beats/min), isoproterenol i.v. was initiated in order to accelerate heart rate aiming at a rate of 90 to 110 beats/min in an effort to further shorten the QT interval. Furthermore, we were prepared to proceed to temporary transvenous cardiac pacing, should the arrhythmic episodes recur. However, no arrhythmic episodes were noticed and the patient remained hemodynamically and electrically stable. Isoproterenol was discontinued three days later with appropriate dosage tapering. The duration of the QTc interval gradually returned to normal within a week.

## Discussion

The present case is the longest known published recording of a self-terminated TdP-VF episode caused by acquired long QT syndrome due to amiodarone [2]. Importantly, in contrast to other reports, the chaotic VF rhythm ceased spontaneously, without a prior conversion to a more organized ventricular rhythm [1-3]. The underlying mechanism for spontaneous arrhythmia termination is unclear. A possible mechanism is that ischemia during VF reduces the tissue excitability through ischemia-dependent hyperkalemia [3]. Hyperkalemia depolarizes the reversal potential of potassium channels IK1 and flattens the restitution curve of the action potential duration, which makes spontaneous arrhythmia termination possible [3].

The combination of QT prolonging drugs with certain predisposing risk factors (bradycardia, recent conversion from atrial fibrillation) probably resulted to this extreme prolongation of QT interval [4,5]. In such clinical scenarios, continuous heart rhythm monitoring and immediate access to defibrillator/temporary pacemaker insertion is necessary [6].

## Conclusions

The combination of QT prolonging drugs with certain predisposing risk factors (bradycardia, recent conversion from atrial fibrillation) could result to extreme QT interval prolongation. In such clinical scenarios, continuous heart rhythm monitoring and immediate access to defibrillator/temporary pacemaker insertion is necessary.
